# Socio-Economic Differences in the Association between Self-Reported and Clinically Present Diabetes and Hypertension: Secondary Analysis of a Population-Based Cross-Sectional Study

**DOI:** 10.1371/journal.pone.0139928

**Published:** 2015-10-14

**Authors:** Gerald Tompkins, Lynne F. Forrest, Jean Adams

**Affiliations:** 1 Health Education North East, Newcastle upon Tyne, United Kingdom; 2 Institute of Health and Society, Newcastle University, Newcastle upon Tyne, United Kingdom; 3 Centre for Diet & Activity Research, MRC Epidemiology Unit, University of Cambridge, Cambridge, United Kingdom; National Institute of Health, ITALY

## Abstract

**Background:**

Diabetes and hypertension are key risk factors for coronary heart disease. Prevalence of both conditions is socio-economically patterned. Awareness of presence of the conditions may influence risk behaviour and use of preventative services. Our aim was to examine whether there were socio-economic differences in awareness of hypertension and diabetes in a UK population.

**Method:**

Data from the Scottish Health Survey was used to compare self-reported awareness of hypertension and diabetes amongst those found on examination to have these conditions, by socioeconomic position (SEP) (measured by occupation, education and income). Odds ratios of self-reported awareness against presence, and the sensitivity, specificity and predictive value of self-reporting as a measure of the presence of the condition, were calculated.

**Results:**

Presence and self-reported awareness of both conditions increased as SEP decreased, on most measures. There was only one significant difference in awareness by SEP once other factors had been taken into account. Sensitivity showed that those in the most disadvantaged groups were most likely to self-report awareness of their hypertension, and specificity showed that those in the least disadvantaged groups were most likely to self-report awareness of its absence. There were few differences of note for diabetes.

**Conclusion:**

We found no consistent pattern in the associations between SEP and the presence and self-reported awareness of hypertension and diabetes amongst those with these conditions. Without evidence of differences, it is important that universal approaches continue to be applied to the identification and management of those at risk of these and other conditions that underpin cardiovascular disease.

## Introduction

Cardiovascular disease (CVD) remains one of the major causes of premature death and disability in the UK [1] and worldwide.[2] Hypertension and diabetes are key risk factors for CVD, with their prevalence (in developed countries) being inversely related to socio-economic position (SEP).[3–5] The Scottish Health Survey (SHS) 2011 [6] reported that almost one in three adults (32.5%) in Scotland had hypertension and 7.6% of adults had diabetes. However, an individual may not be aware that they have these conditions. In the SHS over half of hypertension was untreated and almost one third of diabetes was undiagnosed.[6] Similarly, it has been estimated that there may be 5 million people in England who are unaware of their hypertension.[7]

The concept of health literacy [8] and the links between educational attainment and health behaviours [9–11] both suggest that awareness of conditions that can be symptomless, like hypertension and type 2 diabetes, would increase with SEP. Health literacy has been defined as *“The cognitive and social skills which determine the motivation and ability of individuals to gain access to*, *understand and use information in ways which promote and maintain good health”*.[12] These skills provide tools that enable people to understand messages from health providers, for example about the results of tests indicating a diagnosis. But health literacy is also an asset, increasing the ability to take greater control over life events and the motivation to make positive judgements about health and lifestyle choices.[8–11,13] Poorer health literacy has been associated with poorer understanding of and adherence to medical advice, less use of health services, and worse health outcomes.[13] The ability to identify groups of individuals who are more likely to have low levels of awareness of treatable risk factors should help practitioners better target such groups for secondary prevention–and, if awareness (measured by self-reporting of conditions) is socio-economically patterned, tackle inequalities.

Studies examining the relationship between the self-reported awareness of diabetes or hypertension and SEP do not identify a consistent pattern of association between the two. Where self-reported awareness has been found to increase with SEP, this is often attributed to the better education associated with socio-economic affluence. However, previous studies have been conducted in contexts very different from the UK and generalisability to the UK may be limited.[14–23] The one UK study we are aware of [24] found lower awareness of coronary heart disease (CHD) risk status amongst those in more poorly paid work, but was restricted to a working age population.

The aim of this research was to examine, in a population-representative cohort of adults, whether there were socio-economic differences in self-reported awareness of hypertension and diabetes amongst those in whom these conditions were present on examination. The aim of the study was fulfilled although few significant differences were identified.

## Methods

### Data source, availability and ethics

The SHS is an annual cross-sectional survey [25,26] of a representative sample of the population of Scotland, with the aim of providing a comprehensive profile of the nation’s health. A random sample of private households is selected, from the Postcode Address File, with up to 10 adults and two children being eligible for interview at each sampled address. All those who agree to participate are interviewed, and data is gathered on their health, health-related behaviours, use of healthcare, and socio-demographic factors. In the period covered by this study, a sub-sample of one in six participants was invited to take part in a nurse examination including measurement of height and weight, blood pressure and the collection of urine and blood samples–analysed for, amongst other things, glycated haemoglobin. The SHS thus provides data on both self-reported awareness and clinical measurement of hypertension and diabetes, as well as on SEP, allowing examination of the relationship between SEP and the awareness and presence of these conditions.

A consolidated dataset covering four years of the survey (2008–11) was provided by the SHS team. The data are available to eligible, UK-based researchers, directly from the UK Data Archive (http://discover.ukdataservice.ac.uk/series/?sn=2000047).

The SHS is conducted under ethical approval granted independently from the study. This research did not require additional approval as it was a secondary analysis of anonymised data.

### Variables of Interest

Variables of interest fell into three groups: the awareness of a diagnosis of hypertension and diabetes; the presence of these two conditions; and SEP.

During the interview, participants were asked whether they had ever been diagnosed with high blood pressure or diabetes (no distinction was made between Types 1 and 2) by a doctor: we identified those self-reporting “yes” as aware of their condition. The presence of hypertension was determined at clinical examination as blood pressure of systolic >140 or diastolic >90 mm Hg,[27,28] based on the average of the last two of three readings. Those who were not found to be hypertensive at examination but who were taking anti-hypertensive medication were also identified as hypertensive. The presence of diabetes on examination was defined as glycated haemoglobin of 6.5% or above [29,30] based on a non-fasting sample.

Socio-economic position was measured using individual educational attainment, individual occupational social class and household income. Each was grouped into three levels for analysis. Educational attainment was categorised as “no qualifications”, “school level qualifications” and “post-school level qualifications”. The National Statistics Socio-Economic Classification system [31] was used to classify occupational social class as: routine and manual; intermediate; and higher managerial, administrative and professional, based on the individual’s current or most recent occupation. Household income was based on total household income from all sources, including earnings, pensions and benefits, equivalised for household composition using the McClements’ scale.[26,32] This was grouped into tertiles for analysis.

A number of factors known or hypothesised to confound the relationship between the presence or awareness of the conditions and SEP were included in the analysis. These were: sex, age, ethnicity (white British or other), living with a partner or not,[33] being a current smoker or not, drinking more alcohol than recommended or not, eating less than five portions of fruit and vegetables per day or not, being less physically active than recommended or not, having a body mass index of 25 or more or not, self-reporting less than good general health versus good or very good [34] and living in a rural area or not.[35] Full details of the questions used to ascertain these variables are provided in the SHS documentation.[36]

### Participants in the study

From 2008–11, there were 4273 participants in the SHS who were examined by a nurse, and these people comprised our sample. Of these 4273, those missing information on self-reported awareness, presence of the conditions or the different measures of SEP (e.g. for occupation those who had never worked or did not provide information) were excluded, leaving a sample of 3131 (73.3% of interviewees) for inclusion in the hypertension analyses, and 2761 for diabetes (64.6%). The great majority of attrition was due to absence of information on the presence of diabetes or hypertension–i.e. respondents had declined to give a blood sample or have their blood pressure checked.

The SHS data is provided with a weighting variable that adjusts for selective non-response to both the research interview and nurse visit. The weighting variable was applied in all analyses. This resulted in a final effective sample size of 3035 for hypertension and 2696 for diabetes.

Table 1 shows the characterisitics of this (weighted) population.

**Table 1:** Participant characteristics and demographics.

Variable	Level	Hypertension, N (%)^1^	Diabetes, N (%)^1^
**Measures of SEP**			
Occupational social class	Managerial & professional	1129 (37.2)	1018 (37.8)
	Intermediate	626 (20.6)	542 (20.1)
	Routine and manual	1280 (42.2)	1136 (42.1)
Educational attainment	Post-school qualifications	1280 (42.2)	1117 (41.4)
	School level qualifications	1215 (40.0)	1084 (40.2)
	No qualifications	539 (17.8)	494 (18.3)
Household income	Highest	782 (25.8)	705 (26.2)
	Middle	1295 (42.7)	1136 (42.2)
	Lowest	957 (31.5)	854 (31.7)
**Confounding variables**			
Age	Mean age (median)	47.9 (47.0)	48.0 (47.0)
Sex	Male	1468 (48.4)	1341 (49.7)
Ethnicity	Not white British	185 (6.1)	150 (5.6)
Marital status	Not married/living with a partner	446 (14.7)	406 (15.1)
Smoking	Current smoker	633 (20.9)	664 (24.6)
Alcohol	Drinking more than recommended	1381 (45.5)	1268 (47.0)
Diet	Eating <5 portions of fruit and vegetables per day	2345 (77.3)	2089 (77.5)
Physical activity	Being less physically active than recommended	1869 (61.6)	1622 (60.2)
BMI	BMI of 25 or more	1898 (62.5)	1689 (62.6)
General health	Self-reporting less than good general health	656 (21.6)	563 (20.9)
Access	Living in a remote area	301 (9.9)	276 (10.2)

^1^totals may vary due to application of weighting

### Statistical analysis

Logistic regression models were constructed to compare self-reported awareness amongst those with the conditions across levels of SEP, controlling for confounding factors. The least disadvantaged level of SEP was used as the baseline.

The sensitivity, specificity, positive and negative predictive value (PPV and NPV) [37] of using self-reported awareness as a ‘screening test’ for presence of the conditions were calculated for each of the three levels of each measure of SEP to determine if the accuracy of awareness of the conditions varied by SEP, with 95% confidence intervals being used to determine statistical significance.

## Results

Tables 2 and 3 show the presence and self-reported awareness of hypertension and diabetes by the three measures of SEP. The awareness and presence of both conditions increased significantly as SEP decreased (χ^2^ test for trend), except when presence of diabetes was considered by occupational social class.

**Table 2:** Awareness and presence of hypertension by three markers of socio-economic position, Scottish Health Survey, 2008–11.

Marker of socio-economic position	Level	Total, n (column %)^1^	Awareness of hypertension, n (row %)	Presence of hypertension, n (row %)
Occupational social class	Managerial & professional	1129 (37.2)	235 (20.8)	329 (29.1)
	Intermediate	626 (20.6)	135 (21.6)	202 (32.3)
	Routine and manual	1280 (42.2)	357 (27.9)	460 (35.9)
	χ^2^ (p-value)		16.9 (<0.01)	12.6 (<0.01)
Educational attainment	Post-school qualifications	1280 (42.2)	236 (18.4)	338 (26.4)
	School level qualifications	1215 (40.0)	269 (22.1)	365 (30.0)
	No qualifications	539 (17.8)	222 (41.1)	287 (53.2)
	χ^2^ (p-value)		89.4 (<0.01)	101.1 (<0.01)
Household income	Highest	782 (25.8)	127 (16.2)	178 (22.8)
	Middle	1295 (42.7)	299 (23.1)	427 (33.0)
	Lowest	957 (31.5)	301 (31.5)	385 (40.2)
	χ^2^ (p-value)		55.6 (<0.01)	59.1 (<0.01)
	Total	3035	727 (23.9)	991 (32.7)

^1^totals may vary due to rounding from the application of weighting

**Table 3:** Awareness and presence of diabetes by three markers of socio-economic position, Scottish Health Survey, 2008–11.

Marker of socio-economic position	Level	Total, n (column %)^1^	Awareness of diabetes, n (row %)	Presence of diabetes, n (row %)
Occupational social class	Managerial & professional	1018 (37.8)	41 (4.0)	54 (5.3)
	Intermediate	542 (20.1)	19 (3.5)	33 (6.1)
	Routine and manual	1136 (42.1)	69 (6.1)	82 (7.2)
	χ^2^ (p-value)		5.1 (0.02)	3.4 (0.07)
Educational attainment	Post-school qualifications	1117 (41.4)	35 (3.1)	49 (4.4)
	School level qualifications	1084 (40.2)	41 (3.8)	57 (5.3)
	No qualifications	494 (18.3)	52 (10.5)	63 (12.8)
	χ^2^ (p-value)		32.7 (<0.01)	32.7 (<0.01)
Household income	Highest	705 (26.2)	17 (2.4)	25 (3.5)
	Middle	1136 (42.2)	54 (4.8)	72 (6.3)
	Lowest	854 (31.7)	57 (6.7)	72 (8.4)
	χ^2^ (p-value)		15.4 (<0.01)	15.6 (<0.01)
	Total	2696	129 (4.8)	169 (6.3)

^1^totals may vary due to rounding from the application of weighting

Tables 4 and 5 show the odds of self-reported awareness of hypertension or diabetes where the condition was present, by markers of SEP, before and after adjustment for potential confounding variables. The unadjusted analysis showed some significant differences in awareness of hypertension by SEP. However, most of these associations were extinguished by adjustment. After full adjustment, the only significant difference that remained was lower awareness of hypertension when this was present in those in the intermediate, compared to the managerial and professional, social class. No differences in awareness of diabetes, when it was present, were seen by any marker of SEP, either in the unadjusted or adjusted analysis.

**Table 4:** Socio-economic differences in awareness of hypertension in those in whom it is present, Scottish Health Survey, 2008–11.

Marker of socio-economic position	Level	Awareness of hypertension in those in whom it is present, n (row %)	Unadjusted Odds Ratio (95% CI)	Adjusted Odds Ratio (95% CI)^1^
Occupational social class	Managerial & professional	185 (56.2)	Reference	Reference
	Intermediate	96 (47.8)	0.71 (0.50–1.01)	0.65 (0.44–0.98)
	Routine and manual	269 (58.5)	1.10 (0.82–1.46)	0.87 (0.62–1.21)
Educational attainment	Post-school qualifications	175 (51.6)	Reference	Reference
	School level qualifications	195 (53.3)	1.07 (0.79–1.43)	1.03 (0.73–1.43)
	No qualifications	181 (63.1)	1.59 (1.16–2.19)	0.89 (0.60–1.31)
Household income	Highest	90 (50.3)	Reference	Reference
	Middle	233 (54.6)	1.19 (0.84–1.69)	0.95 (0.64–1.40)
	Lowest	227 (59.0)	1.43 (1.00–2.04)	0.77 (0.51–1.18)

^1^adjusted for smoking, alcohol consumption, diet, level of physical activity, BMI, self-reported general health, access, ethnicity, age, sex and living alone

**Table 5:** Socio-economic differences in awareness of diabetes in those in whom it is present, Scottish Health Survey, 2008–11.

Marker of socio-economic position	Level	Awareness of diabetes in those in whom it is present, n (row %)	Unadjusted Odds Ratio (95% CI)	Adjusted Odds Ratio (95% CI)^1^
Occupational social class	Managerial & professional	33 (60.0)	Reference	Reference
	Intermediate	18 (52.9)	0.74 (0.31–1.76)	0.64 (0.22–1.80)
	Routine and manual	57 (69.5)	1.54 (0.75–3.16)	1.32 (0.55–3.22)
Educational attainment	Post-school qualifications	31 (63.3)	Reference	Reference
	School level qualifications	37 (63.8)	1.00 (0.45–2.20)	0.91 (0.36–2.33)
	No qualifications	39 (61.9)	0.93 (0.43–2.02)	1.16 (0.43–3.18)
Household income	Highest	16 (61.5)	Reference	Reference
	Middle	45 (62.5)	1.02 (0.40–2.59)	0.91 (0.30–2.70)
	Lowest	47 (64.4)	1.14 (0.45–2.91)	1.42 (0.43–4.65)

^1^adjusted for smoking, alcohol consumption, diet, level of physical activity, BMI, self-reported general health, access, ethnicity, age, sex and living alone

Associations between potential confounders and awareness of conditions in those in whom these were present were not the focus of our study but, for all measures of SEP, older age, high BMI and poor self-reported general wellbeing were significantly associated with higher self-reported awareness of hypertension. For diabetes, the only variable significantly associated with greater self-reported awareness was living alone (data not shown).

Tables 6 and 7 set out sensitivity, specificity, PPV and NPV (and 95% confidence intervals) of self-reported awareness for the two conditions, overall and by levels of SEP. The sensitivity showed that overall 55.5% of those with hypertension and 63.3% of those with diabetes self-reported awareness of their condition. Positive predictive values indicated that almost 1 in 4 (24.2%) of those who self-reported hypertension and 1 in 6 (17%) of those who self-reported diabetes did not have the conditions on examination. The specificity showed that of those who did not have hypertension and diabetes, respectively 91.4% and 99.1% correctly identified this, whilst NPV showed that for hypertension 80.9% and for diabetes 97.6% of those who self-reported they had not been diagnosed did not have the condition. Confidence intervals were generally wider for sensitivity and PPV, but narrower for specificity and NPV, and note there was just one significant difference in either sensitivity or PPV for either condition.

**Table 6:** Sensitivity, specificity, positive and negative predictive values of awareness of hypertension by markers of socio-economic position, Scottish Health Survey, 2008–11.

Marker of socio-economic position	Level	Sensitivity, (95% CI)	Specificity, (95% CI)	PPV, (95% CI)	NPV, (95% CI)
All		55.5 (52.4–58.6)	91.4 (90.2–92.6)	75.8 (72.6–78.9)	80.9 (79.3–82.5)
Occupational social class	Managerial & professional	56.2 (50.9–61.6)	93.8 (92.1–95.4)	78.7 (73.5–84.0)	83.9 (81.5–86.3)
	Intermediate	47.8 (40.9–54.7)	91.0 (88.3–93.8)	71.6 (64.0–79.3)	78.6 (75.0–82.2)
	Routine and manual	58.5 (54.0–63.0)	**89.3 (87.2–91.4)**	75.4 (70.9–79.8)	79.3 (76.7–81.9)
Educational attainment	Post-school qualifications	51.6 (46.3–56.9)	93.5 (92.0–95.1)	74.2 (68.6–79.7)	84.3 (82.1–86.5)
	School level qualifications	53.3 (48.2–58.4)	91.2 (89.3–93.1)	72.2 (66.9–77.6)	81.9 (79.5–84.4)
	No qualifications	**63.1 (57.5–68.7)**	**83.8 (79.3–88.3)**	81.5 (76.4–86.6)	**66.7 (61.5–71.9)**
Household income	Highest	50.3 (43.0–57.6)	93.9 (92.0–95.8)	70.9 (63.0–78.8)	86.4 (83.8–89.1)
	Middle	54.6 (49.8–59.3)	92.4 (90.6–94.2)	77.9 (73.2–82.6)	**80.5 (78.1–83.0)**
	Lowest	59.0 (54.2–63.9)	**87.2 (84.5–90.0)**	75.7 (70.8–80.5)	**73.9 (72.6–79.2)**

Bold indicates significantly different from the least disadvantaged level

**Table 7:** Sensitivity, specificity, positive and negative predictive values of awareness of diabetes by markers of socio-economic position, Scottish Health Survey, 2008–11.

Marker of socio-economic position	Level	Sensitivity, (95% CI)	Specificity, (95% CI)	PPV, (95% CI)	NPV, (95% CI)
All		63.3 (56.1–70.6)	99.1 (98.8–99.5)	83.0 (76.5–89.4)	97.6 (97.0–98.2)
Occupational social class	Managerial & professional	60.0 (47.1–73.0)	99.2 (98.6–99.7)	80.5 (68.4–92.6)	97.8 (96.8–98.7)
	Intermediate	52.9 (36.2–69.7)	99.8 (99.4–100)	94.7 (84.7–100)	96.9 (95.5–98.4)
	Routine and manual	69.5 (59.6–79.5)	98.9 (98.2–99.5)	82.6 (73.7–91.6)	97.7 (96.8–98.6)
Educational attainment	Post-school qualifications	63.3 (49.8–76.8)	99.6 (99.2–100)	88.6 (78.0–99.1)	98.3 (97.6–99.1)
	School level qualifications	63.8 (51.4–76.2)	99.6 (99.2–100)	90.2 (81.2–99.3)	98.0 (97.1–98.8)
	No qualifications	61.9 (49.9–73.9)	**97.0 (95.4–98.6)**	75.0 (63.2–86.8)	**94.6 (92.5–96.7)**
Household income	Highest	61.5 (42.8–80.2)	99.9 (99.6–100)	94.1 (82.9–100)	98.6 (97.7–99.4)
	Middle	62.5 (51.3–73.7)	99.2 (98.6–99.7)	83.3 (73.4–93.3)	97.5 (96.6–98.4)
	Lowest	64.4 (53.4–75.4)	**98.6 (97.8–99.4)**	81.0 (71.0–91.1)	96.7 (95.5–98.0)

Bold indicates significantly different from the least disadvantaged level

There was a general trend for sensitivity of awareness of hypertension to increase, and specificity and NPV to decrease, as SEP decreased, but there was no pattern in respect of PPV. Some, scattered, significant differences were seen, as indicated by non-overlap of 95% confidence intervals. Sensitivity was significantly higher amongst the least educated compared to the highest. Specificity was significantly lower for all measures of SEP in the most disadvantaged when compared with the least disadvantaged; however although for all measures of SEP the middle group had higher specificity than the most disadvantaged and lower specificity than the least disadvantaged group, few of these differences were significant.

In respect of diabetes, there was no overall pattern in sensitivity or PPV values by markers of SEP, and there were no significant differences between levels of SEP: note the confidence intervals are wide. Specificity values were high throughout, but were statistically significantly lower for those in the least educated and lowest income groups, compared to the highest. Specificity was also significantly lower in the least educated than the middle level. NPVs were also high throughout, but statistically significantly lower for those in the least educated group compared to the highest and middle groups.

## Discussion

### Statement of principal findings

This is the first UK study we are aware of that has examined the relationship between SEP and self-reported awareness of hypertension or diabetes amongst those with the conditions, in a whole population sample. In general, the presence and self-reported awareness of both conditions increased as SEP decreased, but there were few socio-economic differences in awareness amongst those in whom hypertension or diabetes was present. There was some evidence that those in the most disadvantaged groups were most likely to be aware they had hypertension and those in the least disadvantaged groups were most likely to be aware they did not have hypertension or diabetes.

### Strengths and weaknesses of the study

The use of a population representative cohort increases the generalizability of our findings and our results are likely to apply across the UK. Generalizability to other countries is unknown. Differences in health care and social contexts may limit international comparisons.

Previous studies often focused on a subset of the population–by age,[19,21,22,24,33] work,[24] ethnicity [20] or geography [14]–and their inconsistent findings may reflect this. Other whole population studies found an inverse relationship between SEP and awareness,[18,38] as we did, or no significant results.[23]

We used multiple measures of SEP. Previous studies largely focused on education [14,18,20–23,33,38] though some also reported on income [14,19,21–23] and one on occupation.[33] Our study is unique in examining three measures of SEP, allowing us to consider the impact of different dimensions of SEP.

The variables in the dataset enabled us to identify those whose hypertension was controlled by medication (and include them as hypertensive). The same was not possible for any individuals whose glycated haemoglobin levels were maintained below 6.5% through medication, as this was not provided in the dataset–this may have reduced the numbers for diabetes. The relatively small number of participants with diabetes meant that these analyses lack statistical power (as shown by wide 95% confidence intervals). Future studies using larger cohorts may provide more evidence on this topic.

Our analysis (data not shown) found some differences by SEP between those who were included in the study and excluded because of missing information on self-reported awareness, presence of the conditions or the different measures of SEP. This may have meant that those in the most disadvantaged groups were further under-represented, and weighting may not have compensated sufficiently for this.

Those with cognitive impairment may also have been under-represented: those with severe cognitive impairment may be less likely to have taken part in the study and also be less likely to be aware. Those with mild cognitive impairment may be less likely to be aware and still take part. We did not consider the impact of this.

Many of the variables included as potential confounders were self-reported and may be open to both error and bias [26]. Under-reporting of risk will be independent of the objective presence of the conditions, but may be linked to awareness. As with any epidemiological study, it is also possible that uncontrolled confounding is still present.

There may have been slight under-reporting of awareness, as we relied wholly on the self-report of a diagnosis, and did not explore other markers of awareness, such as reporting use of medication to control their condition.

We were unable to distinguish between type 1 and type 2 diabetes and this may reduce the strength of any effect seen in relation to diabetes: it is unlikely that individuals with T1 diabetes would be unaware of this. Furthermore, the presence of diabetes was determined from a non-fasting sample: this may not represent clinical best practice, but this was the measure provided in the SHS data, and we can anticipate that response rates would be negatively affected by the requirement for a fasting sample.

Our study was limited in the conditions that we were able to examine: we required conditions where the SHS provided data on both the presence (via the nurse examination) and awareness (based on self-report of diagnosis by a doctor). We would, for example, have also examined hypercholesterolemia, but the SHS dataset only included data on its presence, not on awareness.

### Interpretation and implications

We did not find a consistent socio-economic pattern in the relationship between self-reported awareness and the clinical presence of hypertension and diabetes. Known socio-economic differences in health literacy [11,39] mean it might be expected that there would be greater awareness of the conditions amongst the less disadvantaged. However, this was not the case: self-reported awareness of hypertension, and sensitivity of self-reported awareness, increased with prevalence, which increased with disadvantage.

One possible explanation is that those from more disadvantaged backgrounds generally have poorer health [40] and have higher consultation rates for GP appointments.[41] As a result they may be more likely to have had their blood pressure measured and been advised of their hypertension. Alternatively a herd effect may develop: the more common a condition is in a particular community or social group, the more awareness of it grows. Furthermore, those from more disadvantaged socio-economic backgrounds are likely to have lower health expectations and see health status as a result of fate.[42–44] These low expectations may be affirmed by diagnosis, hence higher awareness.

Our findings appear to be at odds with a previous UK study [24] which found evidence of lower awareness of CHD risk amongst those in lower employment grades. The reasons for this difference are not clear, but could include differences in study methodology and population. The previous study included a physical examination, the results of which were sent to participants by letter, informing them of their CHD risk; participants were subsequently asked whether they had ever been informed they were at risk of CHD. The SHS asked for recall of diagnosis by a doctor, which may be more memorable, hence recall being more closely associated with prevalence.

However, the overall sensitivity results showed there were many people who were not aware of their hypertension or diabetes, which has significant implications for their health and wellbeing. As this phenomenon was present across all levels of SEP, efforts to raise awareness of signs and symptoms, and make secondary preventive services available to all,[1] must continue throughout the population–although the higher prevalence in more disadvantaged groups underlines the importance of targeting primary prevention.

The high specificity of self-reporting suggests if someone does not have hypertension or diabetes they are usually aware of this–particularly in less disadvantaged groups.

The fall in NPV as SEP decreased is likely to be explained by the increasing prevalence.[37] However, for the same reason we might have expected differences in PPV by SEP, but this was not generally seen. This may have been a result of a small sample size–confidence intervals were wide–or be explained by factors other than SEP, such as age differences, which we were unable to adjust for when calculating sensitivity, specificity, NPV and PPV.

Furthermore, the PPVs show there were large numbers across all socio-economic groups who reported having the disease when it was not present. This is perhaps understandable for hypertension, which can be reduced through lifestyle changes, but less so in respect of diabetes, which is in effect a lifelong condition once acquired. However, one explanation may be that individuals whose glycated haemoglobin levels were controlled by medication were not identified as having diabetes, unlike the equivalents for hypertension.

Many of these issues could be explored further using qualitative research methods–for example into the factors that influence awareness, and the reasons for people self-reporting having a condition when it is not clinically present.

Further research into awareness of other risk factors, such as hypercholesterolemia and chronic kidney disease, could also be of benefit, for example using data from the Health Survey For England.

Lastly, awareness of the condition is not the same as understanding the consequences for the individual’s health and of the changes in lifestyle (for example) that may be advisable, which may be socio-economically patterned.[8] The communication between clinicians and their patients remains key to influencing behaviour.

## Conclusions

We found no consistent pattern in the associations between socio-economic position and self-reported awareness of the presence of hypertension and diabetes amongst those with these conditions. Without evidence of differences, it is important that universal approaches continue to be applied to the identification and management of those at risk of these and other conditions that underpin cardiovascular disease–but evidence of differences in prevalence underlines the importance of targeted primary prevention activity. We found some evidence that those in the most disadvantaged groups were most likely to be aware they had hypertension and those in the least disadvantaged groups were most likely to be aware they did not have hypertension or diabetes.

Public Health practitioners and clinicians working in primary care must continue to focus on prevention to reduce the presence and progression of conditions such as diabetes and hypertension, and raise awareness amongst all socioeconomic groups of their risk factors and symptoms.
